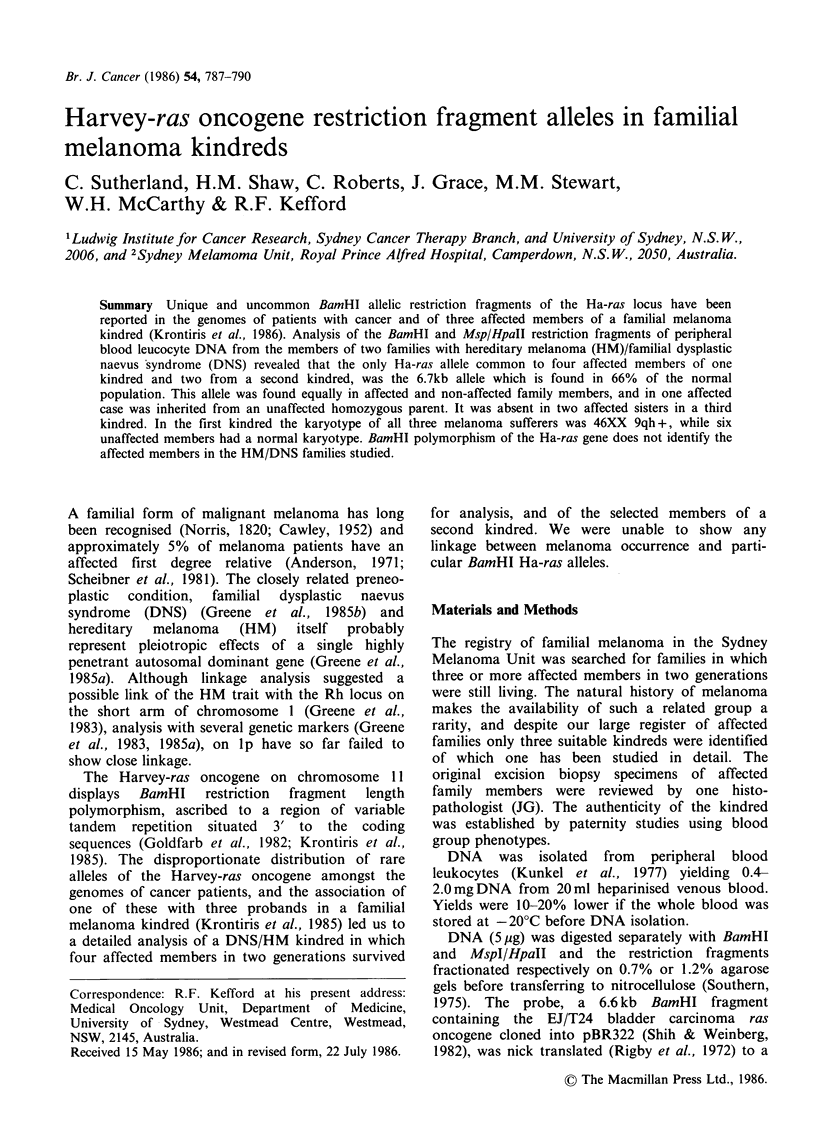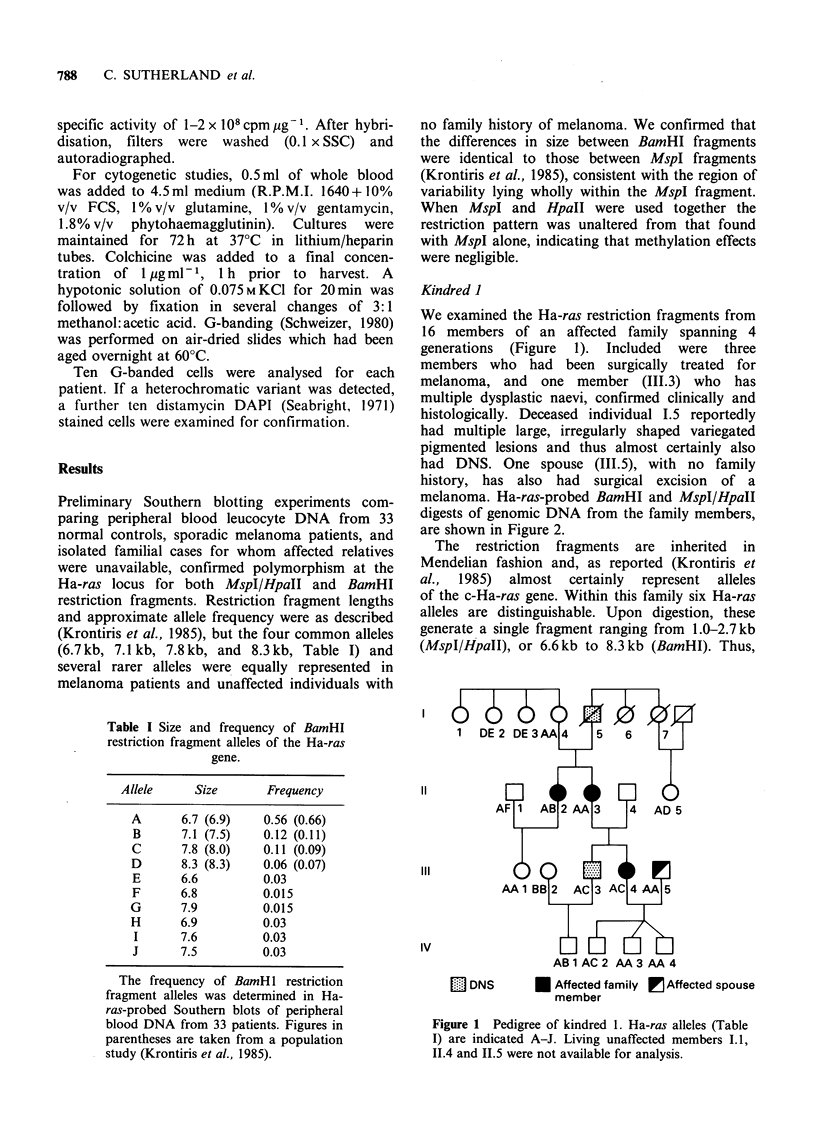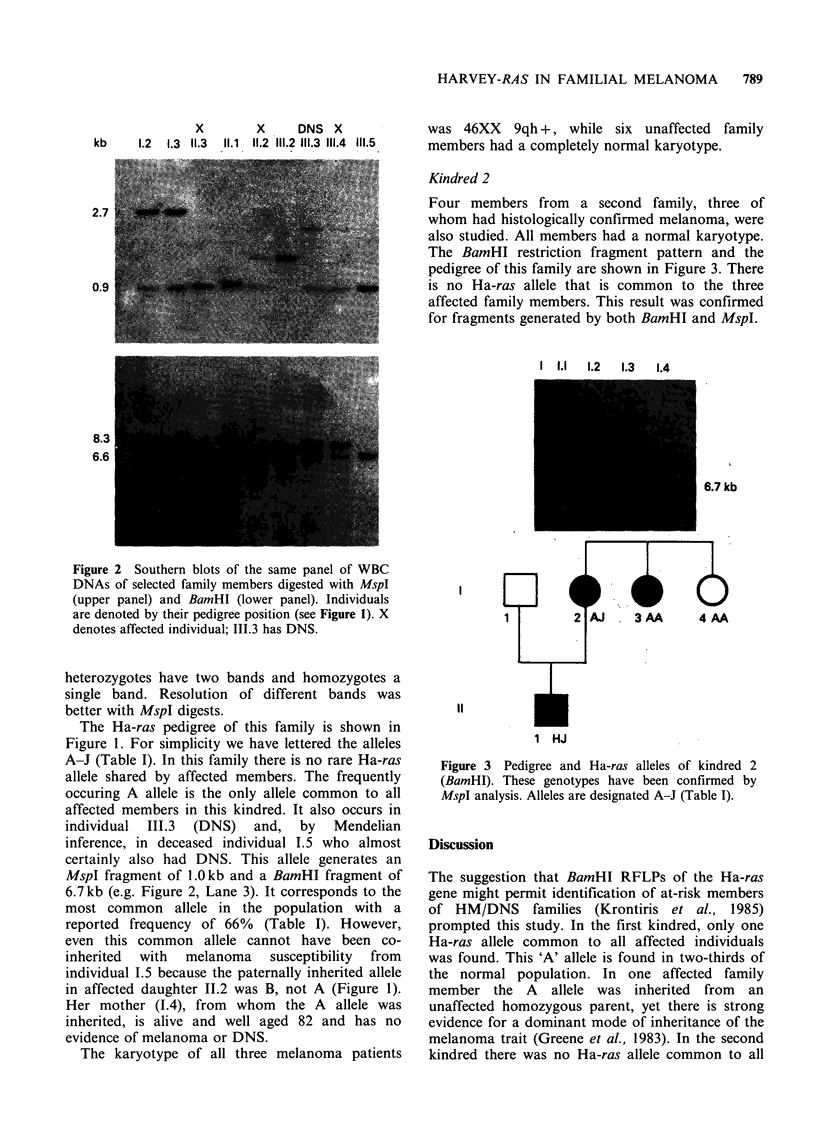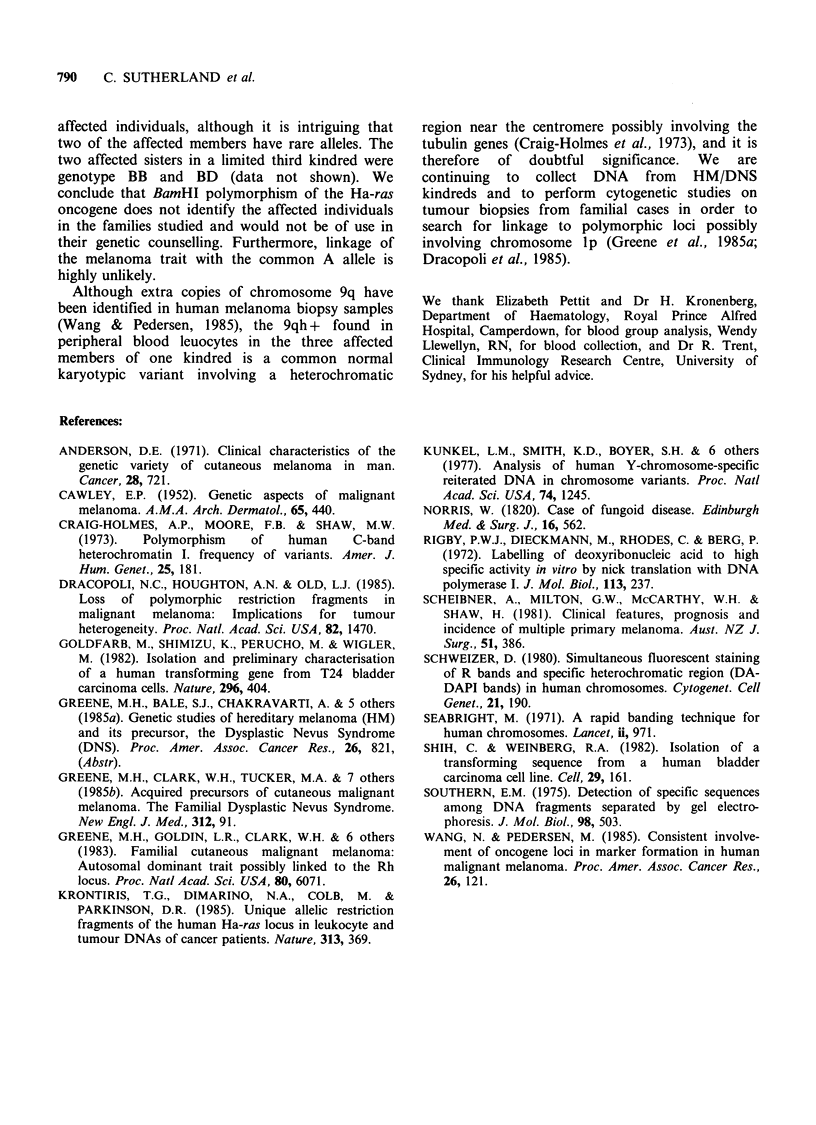# Harvey-ras oncogene restriction fragment alleles in familial melanoma kindreds.

**DOI:** 10.1038/bjc.1986.241

**Published:** 1986-11

**Authors:** C. Sutherland, H. M. Shaw, C. Roberts, J. Grace, M. M. Stewart, W. H. McCarthy, R. F. Kefford

## Abstract

**Images:**


Br. J. Cancer (1986) 54, 787–790

### Harvey-*ras* oncogene restriction fragment alleles in familial melanoma kindreds

C. Sutherland, H.M. Shaw, C. Roberts, J. Grace, M.M. Stewart, W.H. McCarthy & R.F. Kefford

[1]*Ludwig Institute for Cancer Research, Sydney Cancer Therapy Branch, and University of Sydney, N.S.W., 2006, and* [2]*Sydney Melamoma Unit, Royal Prince Alfred Hospital, Camperdown, N.S.W., 2050, Australia.*

**Summary** Unique and uncommon *Bam*HI allelic restriction fragments of the Ha-*ras* locus have been reported in the genomes of patients with cancer and of three affected members of a familial melanoma kindred (Krontiris *et al.*, 1986). Analysis of the *Bam*HI and *Msp/Hpa*II restriction fragments of peripheral blood leucocyte DNA from the members of two families with hereditary melanoma (HM)/familial dysplastic naevus syndrome (DNS) revealed that the only Ha-*ras* allele common to four affected members of one kindred and two from a second kindred, was the 6.7kb allele which is found in 66% of the normal population. This allele was found equally in affected and non-affected family members, and in one affected case was inherited from an unaffected homozygous parent. It was absent in two affected sisters in a third kindred. In the first kindred the karyotype of all three melanoma sufferers was 46XX 9qh+, while six unaffected members had a normal karyotype. *Bam*HI polymorphism of the Ha-*ras* gene does not identify the affected members in the HM/DNS families studied.

A familial form of malignant melanoma has long been recognised (Norris, 1820; Cawley, 1952) and approximately 5% of melanoma patients have an affected first degree relative (Anderson, 1971; Scheibner *et al.*, 1981). The closely related preneoplastic condition, familial dysplastic naevus syndrome (DNS) (Greene *et al.*, 1985*b*) and hereditary melanoma (HM) itself probably represent pleiotropic effects of a single highly penetrant autosomal dominant gene (Greene *et al.*, 1985*a*). Although linkage analysis suggested a possible link of the HM trait with the Rh locus on the short arm of chromosome 1 (Greene *et al.*, 1983), analysis with several genetic markers (Greene *et al.*, 1983, 1985*a*), on 1p have so far failed to show close linkage.

The Harvey-*ras* oncogene on chromosome 11 displays *Bam*HI restriction fragment length polymorphism, ascribed to a region of variable tandem repetition situated 3′ to the coding sequences (Goldfarb *et al.*, 1982; Krontiris *et al.*, 1985). The disproportionate distribution of rare alleles of the Harvey-*ras* oncogene amongst the genomes of cancer patients, and the association of one of these with three probands in a familial melanoma kindred (Krontiris *et al.*, 1985) led us to a detailed analysis of a DNS/HM kindred in which four affected members in two generations survived for analysis, and of the selected members of a second kindred. We were unable to show any linkage between melanoma occurrence and particular *Bam*HI Ha-*ras* alleles.

Correspondence: R.F. Kefford at his present address: Medical Oncology Unit, Department of Medicine, University of Sydney, Westmead Centre, Westmead, NSW, 2145, Australia.

Received 15 May 1986; and in revised form, 22 July 1986.

### Materials and Methods

The registry of familial melanoma in the Sydney Melanoma Unit was searched for families in which three or more affected members in two generations were still living. The natural history of melanoma makes the availability of such a related group a rarity, and despite our large register of affected families only three suitable kindreds were identified of which one has been studied in detail. The original excision biopsy specimens of affected family members were reviewed by one histopathologist (JG). The authenticity of the kindred was established by paternity studies using blood group phenotypes.

DNA was isolated from peripheral blood leukocytes (Kunkel *et al.*, 1977) yielding 0.4–2.0 mg DNA from 20 ml heparinised venous blood. Yields were 10–20% lower if the whole blood was stored at −20°C before DNA isolation.

DNA (5 μg) was digested separately with *Bam*HI and *Msp*I/*Hpa*II and the restriction fragments fractionated respectively on 0.7% or 1.2% agarose gels before transferring to nitrocellulose (Southern, 1975). The probe, a 6.6 kb *Bam*HI fragment containing the EJ/T24 bladder carcinoma *ras* oncogene cloned into pBR322 (Shih & Weinberg, 1982), was nick translated (Rigby *et al.*, 1972) to a

© The Macmillan Press Ltd., 1986.

specific activity of 1–2 $\times 10^8$ cpm $\mu g^{-1}$. After hybridisation, filters were washed (0.1 × SSC) and autoradiographed.

For cytogenetic studies, 0.5 ml of whole blood was added to 4.5 ml medium (R.P.M.I. 1640 + 10% v/v FCS, 1% v/v glutamine, 1% v/v gentamycin, 1.8% v/v phytohaemagglutinin). Cultures were maintained for 72 h at 37°C in lithium/heparin tubes. Colchicine was added to a final concentration of 1 $\mu g\,ml^{-1}$, 1 h prior to harvest. A hypotonic solution of 0.075 M KCl for 20 min was followed by fixation in several changes of 3:1 methanol:acetic acid. G-banding (Schweizer, 1980) was performed on air-dried slides which had been aged overnight at 60°C.

Ten G-banded cells were analysed for each patient. If a heterochromatic variant was detected, a further ten distamycin DAPI (Seabright, 1971) stained cells were examined for confirmation.

### Results

Preliminary Southern blotting experiments comparing peripheral blood leucocyte DNA from 33 normal controls, sporadic melanoma patients, and isolated familial cases for whom affected relatives were unavailable, confirmed polymorphism at the Ha-*ras* locus for both *Msp*I/*Hpa*II and *Bam*HI restriction fragments. Restriction fragment lengths and approximate allele frequency were as described (Krontiris *et al.*, 1985), but the four common alleles (6.7 kb, 7.1 kb, 7.8 kb, and 8.3 kb, Table I) and several rarer alleles were equally represented in melanoma patients and unaffected individuals with no family history of melanoma. We confirmed that the differences in size between *Bam*HI fragments were identical to those between *Msp*I fragments (Krontiris *et al.*, 1985), consistent with the region of variability lying wholly within the *Msp*I fragment. When *Msp*I and *Hpa*II were used together the restriction pattern was unaltered from that found with *Msp*I alone, indicating that methylation effects were negligible.

**Table I** Size and frequency of *Bam*HI restriction fragment alleles of the Ha-*ras* gene.

| *Allele* | *Size* | *Frequency* |
|---|---|---|
| A | 6.7 (6.9) | 0.56 (0.66) |
| B | 7.1 (7.5) | 0.12 (0.11) |
| C | 7.8 (8.0) | 0.11 (0.09) |
| D | 8.3 (8.3) | 0.06 (0.07) |
| E | 6.6 | 0.03 |
| F | 6.8 | 0.015 |
| G | 7.9 | 0.015 |
| H | 6.9 | 0.03 |
| I | 7.6 | 0.03 |
| J | 7.5 | 0.03 |

The frequency of *Bam*H1 restriction fragment alleles was determined in Ha-*ras*-probed Southern blots of peripheral blood DNA from 33 patients. Figures in parentheses are taken from a population study (Krontiris *et al.*, 1985).

*Kindred 1*

We examined the Ha-*ras* restriction fragments from 16 members of an affected family spanning 4 generations (Figure 1). Included were three members who had been surgically treated for melanoma, and one member (III.3) who has multiple dysplastic naevi, confirmed clinically and histologically. Deceased individual I.5 reportedly had multiple large, irregularly shaped variegated pigmented lesions and thus almost certainly also had DNS. One spouse (III.5), with no family history, has also had surgical excision of a melanoma. Ha-*ras*-probed *Bam*HI and *Msp*I/*Hpa*II digests of genomic DNA from the family members, are shown in Figure 2.

The restriction fragments are inherited in Mendelian fashion and, as reported (Krontiris *et al.*, 1985) almost certainly represent alleles of the c-Ha-*ras* gene. Within this family six Ha-*ras* alleles are distinguishable. Upon digestion, these generate a single fragment ranging from 1.0–2.7 kb (*Msp*I/*Hpa*II), or 6.6 kb to 8.3 kb (*Bam*HI). Thus,

**Figure 1** Pedigree of kindred 1. Ha-*ras* alleles (Table I) are indicated A–J. Living unaffected members I.1, II.4 and II.5 were not available for analysis.

**Figure 2** Southern blots of the same panel of WBC DNAs of selected family members digested with *Msp*I (upper panel) and *Bam*HI (lower panel). Individuals are denoted by their pedigree position (see **Figure 1**). X denotes affected individual; III.3 has DNS.

heterozygotes have two bands and homozygotes a single band. Resolution of different bands was better with *Msp*I digests.

The Ha-*ras* pedigree of this family is shown in Figure 1. For simplicity we have lettered the alleles A–J (Table I). In this family there is no rare Ha-*ras* allele shared by affected members. The frequently occuring A allele is the only allele common to all affected members in this kindred. It also occurs in individual III.3 (DNS) and, by Mendelian inference, in deceased individual I.5 who almost certainly also had DNS. This allele generates an *Msp*I fragment of 1.0 kb and a *Bam*HI fragment of 6.7 kb (e.g. Figure 2, Lane 3). It corresponds to the most common allele in the population with a reported frequency of 66% (Table I). However, even this common allele cannot have been co-inherited with melanoma susceptibility from individual I.5 because the paternally inherited allele in affected daughter II.2 was B, not A (Figure 1). Her mother (I.4), from whom the A allele was inherited, is alive and well aged 82 and has no evidence of melanoma or DNS.

The karyotype of all three melanoma patients was 46XX 9qh+, while six unaffected family members had a completely normal karyotype.

*Kindred 2*

Four members from a second family, three of whom had histologically confirmed melanoma, were also studied. All members had a normal karyotype. The *Bam*HI restriction fragment pattern and the pedigree of this family are shown in Figure 3. There is no Ha-*ras* allele that is common to the three affected family members. This result was confirmed for fragments generated by both *Bam*HI and *Msp*I.

**Figure 3** Pedigree and Ha-*ras* alleles of kindred 2 (*Bam*HI). These genotypes have been confirmed by *Msp*I analysis. Alleles are designated A–J (Table I).

### Discussion

The suggestion that *Bam*HI RFLPs of the Ha-*ras* gene might permit identification of at-risk members of HM/DNS families (Krontiris *et al.*, 1985) prompted this study. In the first kindred, only one Ha-*ras* allele common to all affected individuals was found. This 'A' allele is found in two-thirds of the normal population. In one affected family member the A allele was inherited from an unaffected homozygous parent, yet there is strong evidence for a dominant mode of inheritance of the melanoma trait (Greene *et al.*, 1983). In the second kindred there was no Ha-*ras* allele common to all affected individuals, although it is intriguing that two of the affected members have rare alleles. The two affected sisters in a limited third kindred were genotype BB and BD (data not shown). We conclude that *Bam*HI polymorphism of the Ha-*ras* oncogene does not identify the affected individuals in the families studied and would not be of use in their genetic counselling. Furthermore, linkage of the melanoma trait with the common A allele is highly unlikely.

Although extra copies of chromosome 9q have been identified in human melanoma biopsy samples (Wang & Pedersen, 1985), the 9qh+ found in peripheral blood leuocytes in the three affected members of one kindred is a common normal karyotypic variant involving a heterochromatic region near the centromere possibly involving the tubulin genes (Craig-Holmes *et al.*, 1973), and it is therefore of doubtful significance. We are continuing to collect DNA from HM/DNS kindreds and to perform cytogenetic studies on tumour biopsies from familial cases in order to search for linkage to polymorphic loci possibly involving chromosome 1p (Greene *et al.*, 1985*a*; Dracopoli *et al.*, 1985).

We thank Elizabeth Pettit and Dr H. Kronenberg, Department of Haematology, Royal Prince Alfred Hospital, Camperdown, for blood group analysis, Wendy Llewellyn, RN, for blood collection, and Dr R. Trent, Clinical Immunology Research Centre, University of Sydney, for his helpful advice.

**References:**

ANDERSON, D.E. (1971). Clinical characteristics of the genetic variety of cutaneous melanoma in man. *Cancer*, **28**, 721.

CAWLEY, E.P. (1952). Genetic aspects of malignant melanoma. *A.M.A. Arch. Dermatol.*, **65**, 440.

CRAIG-HOLMES, A.P., MOORE, F.B. & SHAW, M.W. (1973). Polymorphism of human C-band heterochromatin I. frequency of variants. *Amer. J. Hum. Genet.*, **25**, 181.

DRACOPOLI, N.C., HOUGHTON, A.N. & OLD, L.J. (1985). Loss of polymorphic restriction fragments in malignant melanoma: Implications for tumour heterogeneity. *Proc. Natl. Acad. Sci. USA*, **82**, 1470.

GOLDFARB, M., SHIMIZU, K., PERUCHO, M. & WIGLER, M. (1982). Isolation and preliminary characterisation of a human transforming gene from T24 bladder carcinoma cells. *Nature*, **296**, 404.

GREENE, M.H., BALE, S.J., CHAKRAVARTI, A. & 5 others (1985*a*). Genetic studies of hereditary melanoma (HM) and its precursor, the Dysplastic Nevus Syndrome (DNS). *Proc. Amer. Assoc. Cancer Res.*, **26**, 821, (*Abstr*).

GREENE, M.H., CLARK, W.H., TUCKER, M.A. & 7 others (1985*b*). Acquired precursors of cutaneous malignant melanoma. The Familial Dysplastic Nevus Syndrome. *New Engl. J. Med.*, **312**, 91.

GREENE, M.H., GOLDIN, L.R., CLARK, W.H. & 6 others (1983). Familial cutaneous malignant melanoma: Autosomal dominant trait possibly linked to the Rh locus. *Proc. Natl Acad. Sci. USA*, **80**, 6071.

KRONTIRIS, T.G., DIMARINO, N.A., COLB, M. & PARKINSON, D.R. (1985). Unique allelic restriction fragments of the human Ha-*ras* locus in leukocyte and tumour DNAs of cancer patients. *Nature*, **313**, 369.

KUNKEL, L.M., SMITH, K.D., BOYER, S.H. & 6 others (1977). Analysis of human Y-chromosome-specific reiterated DNA in chromosome variants. *Proc. Natl Acad. Sci. USA*, **74**, 1245.

NORRIS, W. (1820). Case of fungoid disease. *Edinburgh Med. & Surg. J.*, **16**, 562.

RIGBY, P.W.J., DIECKMANN, M., RHODES, C. & BERG, P. (1972). Labelling of deoxyribonucleic acid to high specific activity *in vitro* by nick translation with DNA polymerase I. *J. Mol. Biol.*, **113**, 237.

SCHEIBNER, A., MILTON, G.W., McCARTHY, W.H. & SHAW, H. (1981). Clinical features, prognosis and incidence of multiple primary melanoma. *Aust. NZ J. Surg.*, **51**, 386.

SCHWEIZER, D. (1980). Simultaneous fluorescent staining of R bands and specific heterochromatic region (DA-DAPI bands) in human chromosomes. *Cytogenet. Cell Genet.*, **21**, 190.

SEABRIGHT, M. (1971). A rapid banding technique for human chromosomes. *Lancet*, **ii**, 971.

SHIH, C. & WEINBERG, R.A. (1982). Isolation of a transforming sequence from a human bladder carcinoma cell line. *Cell*, **29**, 161.

SOUTHERN, E.M. (1975). Detection of specific sequences among DNA fragments separated by gel electrophoresis. *J. Mol. Biol.*, **98**, 503.

WANG, N. & PEDERSEN, M. (1985). Consistent involvement of oncogene loci in marker formation in human malignant melanoma. *Proc. Amer. Assoc. Cancer Res.*, **26**, 121.